# Twenty Years' Experience and Results

**Published:** 1916-03-25

**Authors:** 


### THE SCHOOL DOCTOR.

### Twenty Years' Experience and Results.

The present acute shortage of medical men has led to sharp discussion on the value of the school doctor's work and the extent to which his services are "indispensable." The doctor has now been at work in the schools, in one capacity or another, for over twenty years, and by contrasting the condition of affairs among the elementary school population then with those of the present day the importance of the changes he has been instrumental in effecting become clearly apparent.

The schools of thirty years ago were commonly constructed regardless of the laws of hygiene. Their class-rooms were small, ill-ventilated, and overcrowded by mobs of 80 to 120 children. The heavily ornamented, dark windows shed a dim religious light among the black beams of a vaulted roof, while some of the smaller denominational schools were being conducted in dark cellars or dusty attics, where artificial light was required all day in winter. Naked, flickering gas-jets, sweating walls, and stuffy atmosphere caused headache and nausea to such an extent that Sir J. Crichton Browne reported that he found 52 per cent. of girls and 40 per cent. of boys suffering from headache. Sanitary and cloak-room accommodation was indecently inadequate and often dangerously defective, structural errors being everywhere intensified by a severe economy in cleansing. Floors and windows in some districts were washed once a term, or even less often.

With such an example set by the authorities it is hardly surprising that the personal condition of the children was deplorable. Except in the best schools, cleanliness, as understood now, was a mere dream which the more enthusiastic teachers wore themselves out in trying to realise. The early school doctors learnt to approach their small charges with extreme caution. Lice or ringworm affected most of the children, and in every school great caked masses of scabs, secretion, and vermin could be found on some of the heads. Teachers have related how, after providing fresh garments for some of the more ragged, the school-keeper had to be called in to remove the old clothes with tongs to the boiler fire.

Lack of food had been well recognised, even before the passing of the Elementary Education Act of 1870, as a prevalent evil among children. As a response to the protests of the teachers against the cruelty and absurdity of teaching starving children, a multitude of charitable societies came into existence. But, with the exception of the *Referee* Fund for South London, they were unco-ordinated and inefficient, and wasted huge sums on such absurdities as the wholesale distribution of free soup-tickets or providing starving children with a meal once a week! The number of children totally unprovided for was very serious. In 1889 the London School Board computed that 12.8 per cent. of their scholars were habitually underfed, and of these less than half were relieved by charities. Even in 1904 Dr. Eichholz considered that 16 per cent. of London children required feeding. Until the Provision of Meals Act in 1907 and the coming of the school doctor there was hardly an attempt to deal with malnutrition from a systematic and scientific standpoint.

Many of the evils of school life at this period may fairly be attributed to the teachers' ignorance of hygiene and of the physiological needs of the growing child. Even the college training left them ill-equipped to look after their own health, and often seriously over-strained. Their tendency was to regard recreation hours for themselves or their pupils as so much wasted time. After school was over the head-teacher had to gather her tired pupil-teachers round her for their own instruction before they went home to further study. Small wonder that deafness went undetected, that the cane was the usual treatment for incipient chorea, or that the curriculum sometimes seemed purposely designed to produce myopia and nerve-strain. Fine sewing was universally taught in the infants' department, and, with other kindergarten occupations, such as "designing" and "pricking and embossing," accounted for much damage to the eyes and nerves. The older teachers have warmly testified to the enormous difference that the coming of the school doctor has made to their own health and comfort, and have never failed in generous co-operation.

Medical work in the schools of this country began with a series of small inquiries totally disconnected in time, origin, and purpose. In 1884 a newspaper discussion on nervous over-pressure had led to reports on the health conditions in schools being presented to Parliament by Sir (then Dr.) J. Crichton Browne and Sir J. Fitch. Dr. Priestly Smith directed attention to the onset of myopia among Birmingham pupil-teachers, Dr. Warner's inquiries on nervous signs in children shed light on bad conditions in school life, and evidence from many other quarters demonstrated that a large proportion of children were retarded in school work by unhygienic conditions and by personal defects. During the next ten years the movement for school hygiene spread steadily, and medical officers were appointed in Bradford, Halifax, Burnley, and, later, in Manchester and other smaller towns. Their inquiries, especially those published by Dr. James Kerr, of Bradford, brought to light a mass of defects of vision, hearing, and mental capacity, which deprived many children of education and gravely handicapped them in after life, yet were totally untreated and even unsuspected. Dr. Kerr's discovery of the congenital aphasias (word blindness and deafness) is one example of the many services which medicine has been able to render to education.

It was not, however, any scientific reports or humanitarian feeling for the children of the poor which finally secured public recognition of the need for the school doctor. It was rather due to the scare raised by the enormous number of rejections of recruits for physical defects in the Boer War and the consequent appointment of a Committee on Physical Degeneration. After considering the fragmentary work being done in the way of medical supervision in schools, the Committee reported strongly in favour of some such scheme being made general for the whole country, a recommendation which was put into effect by the Elementary Education (Administrative Provision) Act of 1907. It would be indeed a national calamity if the lessons of the Boer War were forgotten and the school doctor's work was to be stopped on the plea of economy. The improvement he has wrought in the physique of the nation has already justified his existence, and the greater part of his opportunities has yet to be realised.

